# Uric Acid Is Associated with Metabolic Syndrome in Children and Adults in a Community: The Bogalusa Heart Study

**DOI:** 10.1371/journal.pone.0089696

**Published:** 2014-10-24

**Authors:** Dianjianyi Sun, Shengxu Li, Xiaotao Zhang, Camilo Fernandez, Wei Chen, Sathanur R. Srinivasan, Gerald S. Berenson

**Affiliations:** 1 Department of Epidemiology and Biostatistics, School of Public Health, Peking University Health Science Center, Beijing, China; 2 Department of Epidemiology, Tulane University, New Orleans, Louisiana, United States of America; Indiana University Richard M. Fairbanks School of Public Health, United States of America

## Abstract

**Background:**

Elevated serum uric acid (UA) is commonly found in subjects with metabolic syndrome (MetS). This study examined the association of UA with levels of individual MetS components and the degree of their clustering patterns in both children and adults.

**Methods:**

The study sample consisted of 2614 children aged 4–18 years and 2447 adults aged 19–54 years. MetS components included body mass index (BMI), mean arterial pressure (MAP), triglycerides to high-density lipoprotein cholesterol ratio (TG/HDLC), and homeostasis model assessment of insulin resistance (HOMA). Observed/expected (O/E) ratio and intra-class correlation coefficient (ICC) were used as a measure of the degree of clustering of categorical and continuous MetS variables, respectively.

**Results:**

UA was positively and significantly associated only with BMI in children but with all four components in adults. The odds ratio for MetS associated with 1 mg/dL increase of UA was 1.74 (p<0.001) in children and 1.92 (p<0.001) in adults. O/E ratios showed a significant, increasing trend with increasing UA quartiles in both children and adults for 3- and 4-variable clusters with p-values for trend <0.001, except for BMI-MAP-TG/HDLC and MAP-TG/HDLC-HOMA clusters in children and MAP-TG/HDLC-HOMA cluster in adults. ICCs of 3 and 4 components increased with increasing UA quartiles in children and adults.

**Conclusions:**

These results indicate that UA may play a role in the development of MetS in both pediatric and adult populations alike, which may aid in the identification and treatment of high risk individuals for MetS and related clinical disorders in early life.

## Introduction

Uric acid (UA) is the final oxidation product of purine metabolism in humans and higher primates. Since UA has the ability to act as an antioxidant, elevated UA concentration has been considered as a beneficial phenomenon in humans in response to increased oxidative stress in conditions such as cardiovascular disease [1]–[3]. In spite of the antioxidant activity in the extracellular environment, UA has detrimental effects once it enters cells, including vascular smooth muscle cells and adipocytes [4], [5]. Its detrimental impact includes inhibition of endothelial function, induction of platelet aggregation [6], and chronic systemic inflammation [7]. Epidemiologic studies have shown that hyperuricemia is predictive of the development of metabolic syndrome (MetS) [8]–[14], obesity [15], [16], diabetes [17], [18], hypertension [15], [19], [20], kidney disease [18] and cardiovascular disease [18], [21].

MetS, a concurrence of disorders including obesity, insulin resistance, dyslipidemia and hypertension, has gained importance because of its association with subsequent development of cardiovascular disease and type 2 diabetes [22], [23]. MetS is highly prevalent in adults [22], [24], and also occurs commonly in children [25]–[27]. Furthermore, there has been a large body of evidence from both cross-sectional and longitudinal studies showing that hyperuricemia is associated with MetS in both children and adults in multiple ethnic populations [8]–[14], [26], [28], [29]. Although the association between UA and adverse levels of individual MetS components has been extensively investigated, the impact of UA on the degree of clustering of multiple MetS components is incompletely understood, especially in the pediatric age. The objective of the present study is to examine the association between UA and MetS in terms of levels of individual components and the degree of their clustering patterns in both children and adults enrolled in the Bogalusa Heart Study, a long-term community-based biracial (black-white) epidemiologic study of cardiovascular risk factors of children that began in 1973 [30].

## Methods

### Ethics Statement

All subjects in this study gave informed written consent at each examination, and for those under 18 years of age, the written consent of a parent/guardian was obtained. Study protocols were approved by the Biomedical committee of the Tulane University Institutional Review Board.

### Study cohort

In the community of Bogalusa, LA, a survey of 3326 school age children was conducted during 1987–1988, and eight surveys of 3490 adults in total were conducted during 1988–2010 for cardiovascular risk factors and UA. Individuals who were under treatment for hypertension, dyslipidemia or diabetes (5 children, 466 adults) or those who had non-fasting blood samples (476 children, 226 adults) or missing values of any study variables (231 children, 351 adults) were excluded. The final study sample for this report consisted of 2614 children aged 4–18 years and 2447 adults aged 19–54 years.

### General examination

As described elsewhere, all examinations were conducted by trained examiners who followed rigid protocols and procedures [30]. Participants were instructed to fast for 12 to 14 hours before the examination and compliance was ascertained by interview on the morning of screening. Replicate measurements of height and weight were made, and the mean values were used for analysis. Body mass index (BMI, weight in kilograms divided by the square of the height in meters) was used as a measure of obesity. Blood pressures were measured between 8:00 AM and 10:00 AM on the right arm of subjects in a relaxed, sitting position by 2 trained observers (3 replicates each). Systolic and diastolic (4th Korotkoff phase for children and 5th Korotkoff phase for adults) blood pressures were recorded using a mercury sphygmomanometer. The mean values of the 6 readings were used for analysis. Mean arterial pressure (MAP) was calculated as MAP = diastolic blood pressure+1/3 pulse pressure.

### Laboratory Analysis

From 1987 to 1996, cholesterol and triglycerides (TG) levels were determined by enzymatic procedures on the Abbott VP instrument (Abbott Laboratories, Chicago, IL) and on the Hitachi 902 Automatic Analyzer (Roche Diagnostics, Indianapolis, IN) after 1996. Serum lipoprotein cholesterols were analyzed by using a combination of heparin-calcium precipitation and agar-agarose gel electrophoresis procedures. Both chemical and enzymatic procedures met the performance requirements of the Lipid Standardization Program of the Centers for Disease Control and Prevention, which has routinely monitored the precision and accuracy of cholesterol and triglyceride measurements since 1973. Measurements on CDC-assigned quality control samples showed no consistent bias over time within or between surveys.

From 1981 to 1991, plasma glucose was measured by a glucose oxidase method using a Beckman Glucose Analyzer (Beckman Instruments, Palo Alto, CA). Since then, it has been measured enzymatically as part of a multichemistry (SMA20) profile. Plasma immunoreactive insulin levels were measured by a commercial radioimmunoassay kit (Phadebas, Pharmacia Diagnostics, and Piscataway, NJ). An index of insulin resistance was calculated according to the homeostasis model assessment (HOMA) of insulin resistance formula: HOMA = fasting insulin (µU/mL)×fasting glucose (mmol/L)/22.5. Serum UA levels were determined as part of SMA20 by the multichannel Olympus Au-5000 analyzer (Olympus) with the uricase method.

### Statistical Analyses

All data management and analyses were conducted using SAS 9.3 (SAS Institute, Cary, NC). TG/HDLC and HOMA were log-transformed to improve their normality and used for subsequent association analyses. Analyses of covariance were performed using generalized linear models to test differences in study variables between blacks and whites and between males and females. Multivariable linear regression and multivariable logistic regression models were used to examine the association between UA and individual MetS components as continuous variables and the association between UA and MetS as a dichotomous variable, respectively.

Since standard cutoff points for BMI, MAP, TG/HDLC and HOMA are not available for children, the adverse levels of these components were defined as >75th percentile specific for age, race and gender for both children and adults. MetS was defined as the presence of 3 or more MetS components with adverse levels as used in our previous report [25], [31].

The ratio of observed number to expected number (O/E) was applied to estimate the degree of clustering of adverse levels (disorders) of MetS components as dichotomous variables. The values of individual MetS components were first adjusted for age by regression residual analyses, and then the adverse levels were defined as values above 75th percentiles by race and gender groups. The observed number (O) is the number of individuals with 3 or 4 disorders observed in the sample; the expected number (E) was calculated by multiplying the sample size by 0.25^3^*(1−0.25) for 3-variable clusters and by 0.25^4^ for the 4-variable cluster. Significance tests for O/E ratios were performed using a generalized one-sample binomial test when E> = 5 and using the Poisson distribution when E<5 [32].

Intra-class correlation (ICC) based on standardized variables was used in the present study as another statistical approach to evaluate the degree of clustering of continuous risk variables. In order to make the 4 variables to have a uniform unit, the values of MetS components were adjusted for age by regression residual analyses and then standardized with Z-transformation (mean = 0, SD = 1) by race and gender groups prior to ICC analysis. The standardized variables (Z-scores) were used in one-way analysis of variance, and the ICC coefficient was calculated by the expression below [25], [31], [33].
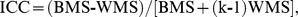
where k is the number of study variables; BMS is between-subject mean square; WMS is within-subject mean square. The test that ICC is different from zero is provided by calculating F = BMS/WMS on (n−1) and n(k−1) degrees of freedom, where n is the total sample size.

## Results

Table 1 shows mean levels and SD of continuous study variables of children and adults by race and gender. The mean levels of study variables were compared between race-gender groups, adjusting for age (except age itself). Among children, there were significant race differences in UA and log-TG/HDLC (whites>blacks) in both boys and girls, and gender differences in UA (boys>girls) and log-TG/HDLC (girls>boys). Log-HOMA showed a significant gender difference (girls>boys) in both races, but race difference was not significant; girls had significantly higher levels of BMI and MAP than boys among whites only. Among adults, UA showed a significant gender difference (males>females) for both races, but the race difference was not significant. The four MetS components differed significantly between gender and race groups except for the race difference in BMI and Log-HOMA among males.

**Table 1:** Characteristics (Mean±SD, %) of study variables by race and gender.

	Whites		Blacks		P for Race Difference	
	Males	Females	Males	Females	Males	Females
Children (4–18 years)						
N	804	773	529	508		
Age (years)	10.8±3.5	10.8±3.5	11.2±3.6	11.2±3.6	0.062	0.029
UA (mg/dL)	4.5±1.4	4.1±1.1**	4.1±1.4	3.6±1.0**	<0.001	<0.001
BMI (kg/m^2^)	19.0±4.2	19.5±4.7*	19.3±4.6	19.5±4.7	0.813	0.205
MAP (mmHg)	72.1±9.0	73.0±9.2*	73.2±9.8	73.2±10.5	0.235	0.278
Log-TG/HDLC	0.21±0.55	0.32±0.53**	−0.06±0.45	0.03±0.43**	<0.001	<0.001
Log-HOMA	0.42±0.55	0.60±0.60**	0.44±0.60	0.62±0.66**	0.923	0.796
MetS (%)	11.7	13.7	10.2	11.2	0.399	0.190
Adults (19–54 years)						
N	743	939	329	436		
Age (years)	33.2±8.8	32.7±8.9	31.3±9.1	30.4±8.5	0.002	<0.001
UA (mg/dL)	6.1±1.3	4.2±1.1**	6.0±1.5	4.2±1.2**	0.460	0.991
BMI (kg/m^2^)	27.6±5.6	26.4±6.8**	27.2±6.7	29.1±7.9**	0.787	<0.001
MAP (mmHg)	90.5±9.0	84.9±8.3**	92.2±12.2	87.0±10.3**	<0.001	<0.001
Log-TG/HDLC	1.06±0.80	0.68±0.66**	0.57±0.71	0.30±0.51**	<0.001	<0.001
Log-HOMA	0.70±0.68	0.58±0.64**	0.59±0.73	0.79±0.67**	0.036	<0.001
MetS (%)	12.7	15.0	15.2	10.6	0.260	0.025

Gender difference within racial groups: * p<0.05, ** p<0.01;

UA = uric acid; BMI = body mass index; MAP = mean arterial pressure; TG/HDLC = ratio of triglycerides to HDL cholesterol; HOMA = homeostatic model assessment of insulin resistance.

Table 2 presents linear regression coefficients of MetS components on UA in children and adults by race and gender and in the total sample. Since BMI is known to be strongly associated with UA and other MetS components, BMI was included as a confounder in regression models for MAP, log-TG/HDLC and Log-HOMA in addition to age. Standardized regression coefficients are also presented in Table 2 for the purpose of comparing the strength of the association. Higher UA was most strongly associated with higher values of BMI consistently across all race and gender groups for both children and adults; whereas UA was associated with other MetS components in selected subgroups. In the total sample, UA was positively, significantly associated with all the four MetS components in adults; however, UA was significantly associated with BMI and log-HOMA in children. Of note, the association between UA and individual MetS components was consistently stronger in adults than in children based on the standardized regression coefficients.

**Table 2:** Unstandardized (standardized) regression coefficient of MetS components on uric acid in children and adults by race and gender, adjusted for age and BMI.

Dependent Variables	Whites		Blacks		All
	Males	Females	Males	Females	
Children (4–18 years)					
BMI (kg/m^2^)†	1.01 (0.34)***	1.31 (0.31)***	1.31 (0.39)***	1.22 (0.27)***	1.10 (0.32)***
MAP (mmHg)	−0.06 (−0.01)	0.15 (0.02)	−0.12 (−0.02)	0.87 (0.09)*	0.11 (0.02)
Log-TG/HDLC	0.003 (0.009)	0.002 (0.004)	−0.005 (−0.014)	0.030 (0.074)	0.014 (0.034)
Log-HOMA	−0.015 (−0.038)	0.055 (0.103)***	−0.003 (−0.008)	0.084 (0.134)***	0.019 (0.041)*
Adults (19–54 years)					
BMI (kg/m^2^)†	1.38 (0.33)***	2.59 (0.44)***	1.29 (0.28)***	2.14 (0.33)***	1.87 (0.43)***
MAP (mmHg)	0.53 (0.08)*	0.10 (0.01)	0.37 (0.04)	1.12 (0.13)**	0.53 (0.08)***
Log-TG/HDLC	0.069 (0.115)***	0.091 (0.159)***	0.124 (0.255)***	0.068(0.163)***	0.089 (0.189)***
Log-HOMA	0.032 (0.063)*	0.061 (0.109)***	−0.027 (−0.053)	0.036 (0.067)	0.030 (0.069)***

† , BMI was not included in the model as a covariate.

Regression coefficient different from 0: * p<0.05, ** p<0.01, *** p<0.001.

Table 3 presents odds ratio (OR) between MetS and UA derived from multivariable logistic regression models in children and adults by race and gender and in the total sample for both age groups. UA showed significant and consistent associations with MetS (OR = 1.53–2.59, p<0.01) across race-gender groups, adjusting for age in both children and adults. In the total sample for both ages, the prevalence of MetS was 11.9% in children and 13.4% in adults. Females were more likely to have MetS (OR = 1.55, p<0.01 for children; OR = 3.65, p<0.01 for adults). The risk of MetS associated with an increase of one unit (mg/dL) of UA was 1.74 times higher in children and 1.92 times higher in adults.

**Table 3:** Odds ratios (95% confident interval) of uric acid for MetS in children and adults by race and gender.

Independent Variable	Whites		Blacks		All
	Males	Females	Males	Females	
Children (4–18 years)					
Black Race	—	—	—	—	1.07 (0.83, 1.39)
Female Gender	—	—	—	—	1.55 (1.20, 2.00)
Age (years)	0.97 (0.91, 1.04)	1.02 (0.96, 1.08)	0.84 (0.76, 0.93)	1.00 (0.92, 1.09)	0.96 (0.93, 1.00)
Uric acid (mg/dL)	1.53 (1.29, 1.82)	1.80 (1.50, 2.17)	2.15 (1.69, 2.75)	2.17 (1.66, 2.85)	1.74 (1.58, 1.92)
Adults (19–54 years)					
Black Race	—	—	—	—	0.87 (0.66, 1.14)
Female Gender	—	—	—	—	3.65 (2.66, 5.00)
Age (years)	1.02 (0.99, 1.04)	1.00 (0.97, 1.02)	1.00 (0.97, 1.04)	0.98 (0.94, 1.01)	1.00 (0.99, 1.01)
Uric acid (mg/dL)	1.79 (1.52, 2.11)	2.59 (2.14, 3.12)	1.56 (1.26, 1.94)	1.71 (1.35, 2.18)	1.92 (1.74, 2.12)

**Figure 1:**
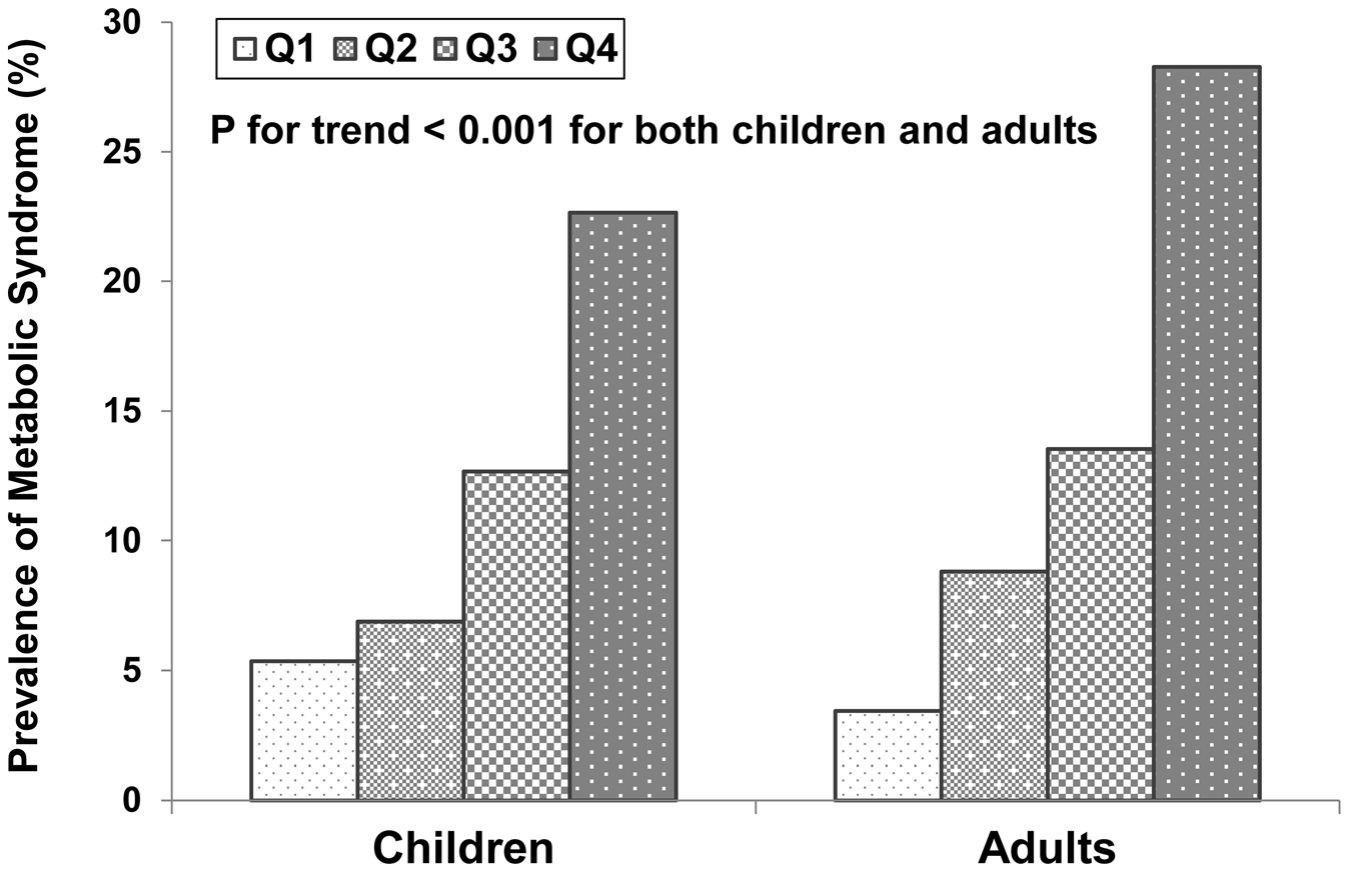
Race-, gender- and age-adjusted prevalence of metabolic syndrome (MetS) by uric acid quartiles (Q).

Figure 1 illustrates the relationship between the prevalence of MetS and quintiles of UA specific for race, gender and age in the total sample for both children and adults. The prevalence of MetS significantly increased with increasing quartiles of UA in children and adults (p for trend <0.001). The prevalence of MetS did not show significant gender difference in both children and adults.

**Figure 2:**
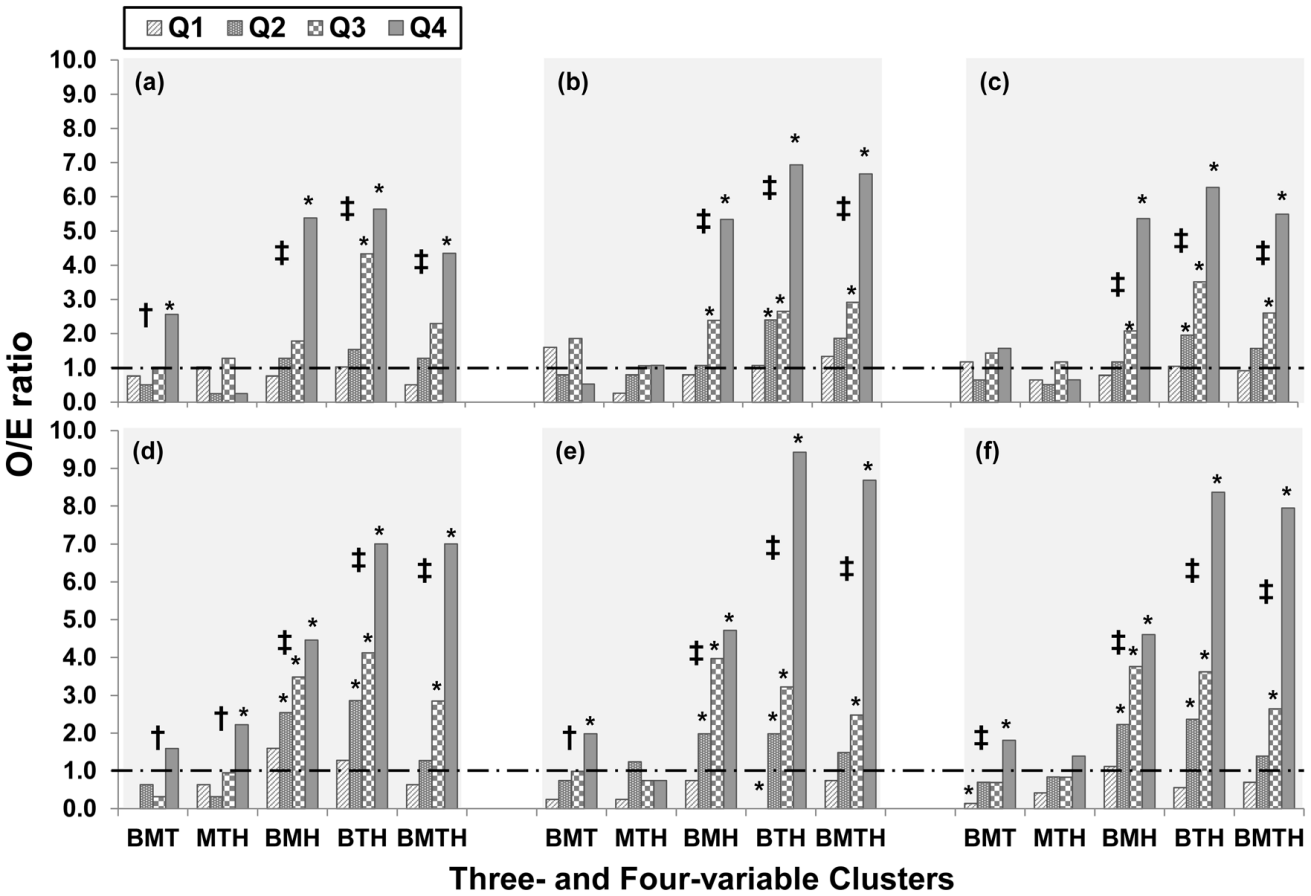
Observed to expected (O/E) ratio of clustering patterns of MetS components by uric acid quartiles (Q) in children and adults. (a) boys; (b) girls; (c) all children; (d) males adults; (e) female adults; (f) all adults. BMT = BMI, MAP and TG/HDLC; MTH = MAP, TG/HDLC and HOMA; BMH = BMI, MAP and HOMA; BTH = BMI, TG/HDLC and HOMA; BMTH = BMI, MAP, TG/HDLC and HOMA. *, O/E ratios are significantly different from 1 (p<0.05). †, p for trend <0.05. ‡, p for trend <0.001.

Four MetS components produced four combinations of 3 variables and one combination of all 4 variables (Figure 2). O/E ratios in children significantly increased with increasing UA quartiles for BMI-MAP-HOMA (BMH), BMI-TG/HDLC-HOMA (BTH) and BMI-MAP-TG/HDLC-HOMA (BMTH) clusters in boys, girls and the total sample (p for trend <0.001); adults showed similar trends in BMI-MAP-TG/HDLC (BMT), BMH, BTH and BMTH clusters for males, females and the total sample (p for trend <0.05). Of interest, 3-variable clusters involving both BMI and HOMA showed higher values of O/E ratios and greater trends than other 3-variable clusters involving either BMI or HOMA, but not both.

**Figure 3:**
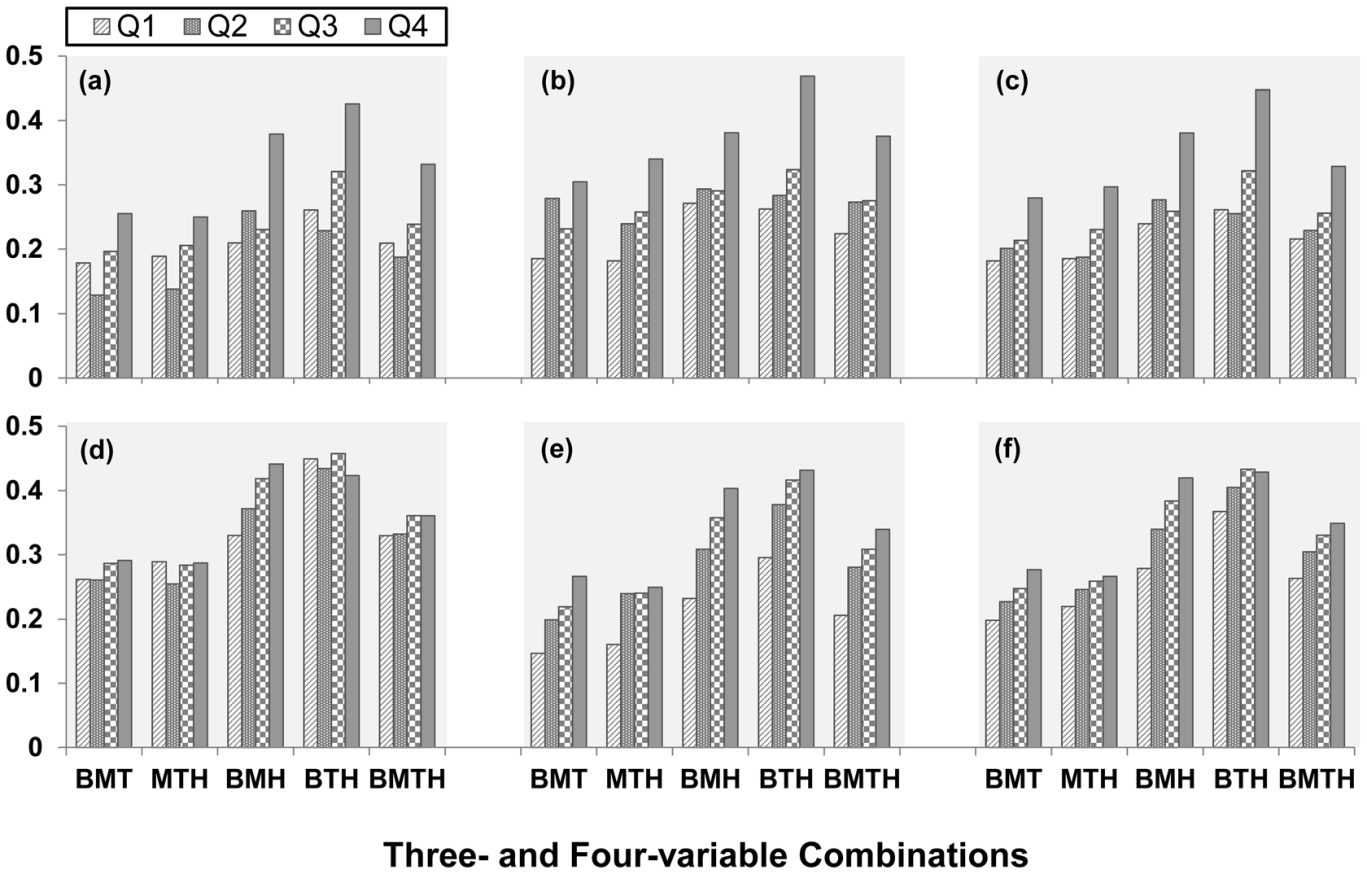
Intra-class correlation of MetS components by uric acid quartiles (Q) in children and adults. (a) boys; (b) girls; (c) all children; (d) male adults; (e) female adults; (f) all adults. BMT = BMI, MAP and TG/HDLC; MTH = MAP, TG/HDLC and HOMA; BMH = BMI, MAP and HOMA; BTH = BMI, TG/HDLC and HOMA; BMTH = BMI, MAP, TG/HDLC and HOMA. All intra-class correlation coefficients are significantly greater than 0 (p<0.001).

In Figure 3, ICC coefficients among 3 and 4 components as continuous variables were all significantly greater than zero (p<0.001) both in children and in adults. Although a standard significance test for the trend of ICC coefficients is not available, the ICC coefficients showed clear increasing trends with increasing quintiles of UA in both children and adults, adjusted for race, gender and age. With respect to the degree of clustering by gender groups, children had increasing trends in ICC coefficients with increasing UA levels in both boys and girls; adults showed strikingly increased ICC coefficients with increasing UA quartiles consistently for all clusters in females; however, no such trends in ICC coefficients were noted in males except for the BMH cluster.

## Discussion

Although UA is not part of any definition of the MetS, many epidemiologic studies have shown that hyperuricemia is associated with MetS in multiple ethnic populations. Most previous studies have reported the association between UA and levels of individual MetS components and their clustering in adults [8]–[12], [14] as well as in children [26], [28], [29]. The present study found that the association between UA and levels of individual MetS components was stronger in adults than in children, with UA showing the strongest and most consistent association with BMI in all race-gender subgroups; however, UA had a more consistent influence on the degree of clustering of 3- and 4-variable clusters in children than in adults as measured by O/E ratio and ICC. The results from the current study suggest that elevated UA plays a key role in the pathogenesis of MetS and its influence begins in childhood.

It has been well documented that MetS begins in childhood, and multiple MetS risk variables interact with each other and persist (track) from childhood into adulthood [25], [27], [34]. Furthermore, a number of studies have shown strong associations of concentrations of UA with MetS or its components, primarily in adults [8]–[12], [14]; data are relatively limited about the association between UA and MetS in children [26], [28], [29]. In a nationally representative sample of 1,370 US children and adolescents aged 12–17 years from NHANES 1999–2002, a graded positive association between serum UA quartiles and the prevalence of MetS was noted. A prevalence of MetS of 21.1% in children with the highest quartile of UA was observed [26]. In our analysis, we found a significant increasing trend in the prevalence of MetS with increasing UA quartiles in a community-based sample of children aged 4–18 years. The prevalence of MetS was 22.7% in children with the highest UA quartile in our sample, which was similar to the prevalence noted in the sample from NHANES [26] in spite of different definitions of the MetS. In addition, we found that UA also showed a strong and consistent association with the degree of clustering of MetS components in children in terms of O/E ratio and ICC coefficients.

Since the risk variables and criteria to diagnose MetS that have been used for adults [24] are not applicable to children, and a standard pediatric definition of MetS is not yet available [35], we used BMI, MAP, TG/HDLC and HOMA as components to define the syndrome for both children and adults. These four components and the cut-off points (>75th percentile specific for age, gender and race) used in the current study are consistent with those used in our previous studies [25], [36]. In the present analysis, we found that elevated UA levels were significantly associated with increased prevalence of MetS in both children and adults as shown in Figure 1. Further, the significantly graded trend of O/E ratios with increasing UA levels (Figure 2) indicated that elevated UA is associated not only with the higher prevalence of adverse levels of individual MetS components, but also with the degree of clustering of multiple MetS components measured as O/E ratios in both children and adults. The observed number of subjects with four disorders was 5.5 and 8.0 times the expected number in children and adults, respectively, with the top quartile of UA. In particular, children showed a stronger UA-clustering association (Figure 2) than the UA-level association (Table 2).

Since, unlike O/E ratios, the ICC involves continuous variables that obviate problems associated with the definition of MetS using cutoff points of the components, we used ICC among the four component variables as another quantitative approach measuring the degree of clustering of components. As shown in Figure 3, ICC coefficients were all significantly greater than zero in children and adults. The ICC coefficients showed clear increasing trends with increasing quintiles of UA for the combinations of 3 and 4 components in the total sample of both children and adults, although the trends in adults were not so impressive compared with those in children. In contrast with the difference between UA-clustering and UA-level associations as mentioned above, adults showed a weaker UA-clustering association (Figure 3) than the UA-level association (Table 2). This discrepancy in the UA-clustering associations between children and adults measured as O/E ratios and ICC coefficients may be explained by the threshold effect of UA and/or the MetS components on the degree of clustering patterns. A number of studies have shown that the UA level and its association with MetS risk variables are affected and confounded more commonly in adults by other factors such as impaired renal function, medications, lifestyles, diabetes and cardiovascular disease [14], [37]–[40]. Greater cumulative burden of these factors in adults may reduce the correlation among the continuous MetS components in individuals who have values above the cutoff of 75^th^ percentile (threshold) we used in the current study. Another possible explanation is the gender-diversity of the association between UA and MetS.

It has been well documented that serum UA levels were lower in women than in age-matched men [39]–[41] due to the potential productive effect of estrogens by the postsecretory tubular reabsorption of UA [42], [43]. Despite the lower levels of UA, women have shown a consistently stronger association between UA and MetS than men [8], [40], [44]. In a cross-sectional study of 8,144 Japanese individuals, the odds ratio for MetS associated with top quartile of UA was 4.2 in women and 2.0 in men using the bottom quartile of UA as a reference [40]. In the current study, levels of serum UA were significantly lower in females than in males for both children and adults (Table 1); as shown in Table 3, odds ratios for MetS associated with UA were consistently higher in females than those in males across all race-gender subgroups in both children and adults, especially in white adults. With respect to the degree of clustering in gender groups, we found that both O/E ratios and ICC coefficients showed consistent, striking increasing trends with UA quartiles in female adults; however, no such trends in ICC coefficients were noted in male adults. Further studies in depth are needed to investigate the gender-specific association between UA and degree of clustering of MetS componets in various age groups.

In summary, we used a community-based sample and quantitative statistical approaches to examine the possible role of UA in the development of MetS in the present study. We found a positive, significant association between serum UA and MetS in children and adults. Elevated UA was more strongly associated with adverse levels of individual MetS components in adults; while increased UA levels were more strongly associated with the degree of clustering in children as measured by O/E ratio and ICC coefficients. Further research is needed for the gender-divergence in the association between serum UA and MetS. Our findings have implications for identification of a juvenile group at a higher risk of MetS and potential for treatment to prevent MetS in early life and thus reduce the risk of cardiovascular disease.
